# Supplementary material for: Morphological changes in amblyopic eyes in choriocapillaris and Sattler’s layer in comparison to healthy eyes, and in retinal nerve fiber layer in comparison to fellow eyes through quantification of mean reflectivity: A pilot study
Source: PLoS One. 2021 Aug 6;16(8):e0255735. doi: 10.1371/journal.pone.0255735 (PMC8345865; doi:10.1371/journal.pone.0255735)
Supplement: S1 File — (DOCX) [file pone.0255735.s001.docx]

**S1 File**

All of these SX figures are mentioned in the materials and methods section to better clarify our method of measurement.

Table 1 : the SX fig. mentioned in the materials and methods part.

**(S1 Fig. Delineating the scale bar, S2 Fig. Setting the scale, S3 Fig. Marking the center, S4 Fig. Measuring distance away from center, S5 Fig. Borders for ROIs#1-6, S6 Fig. ROI#1, S7 Fig. ROI#2, S8 Fig. ROI#3, S9 Fig. ROI#4, S10 Fig. ROI#5, S11 Fig. ROI#6, S12 Fig. Borders for ROIs#7-11, S13 Fig. ROI#7, S14 Fig. ROI#8, S15 Fig. ROI#9, S16 Fig. ROI#10, S17 Fig. ROI#11, S18 Fig. ROI#12.)**

| 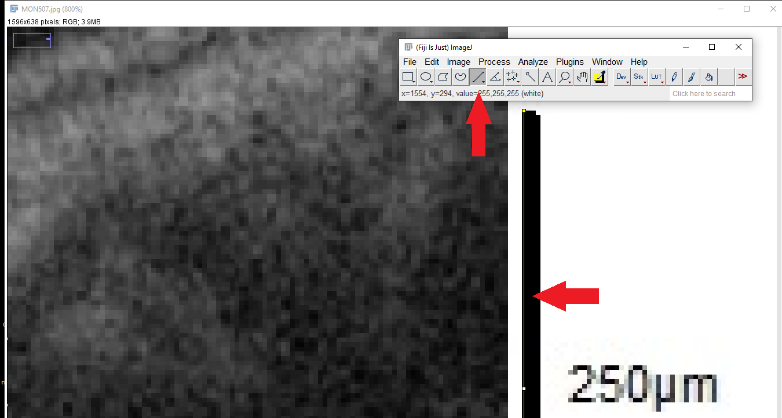  S1_Fig | 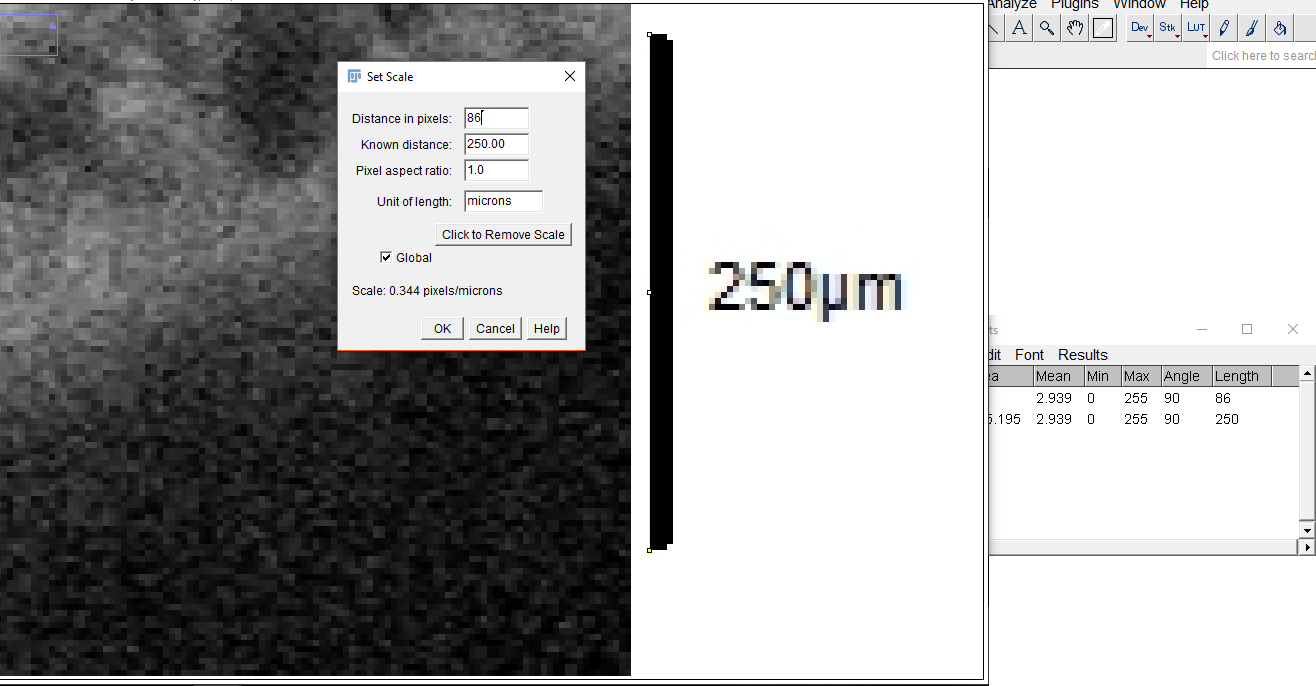  S2_Fig |
| --- | --- |
| 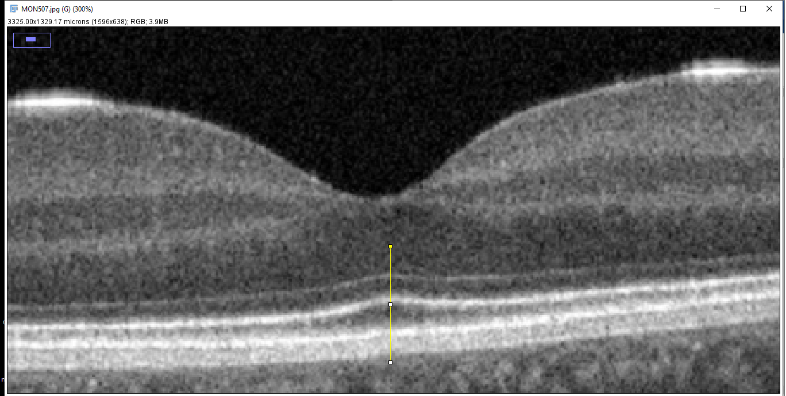  S3_Fig | 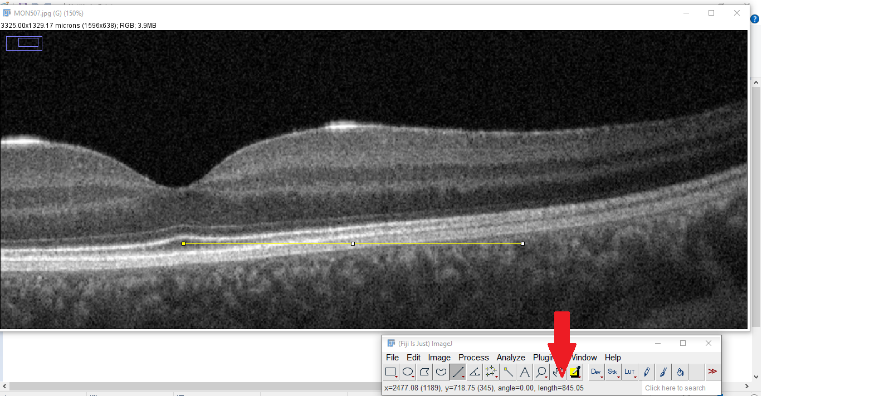  S4_Fig |
| 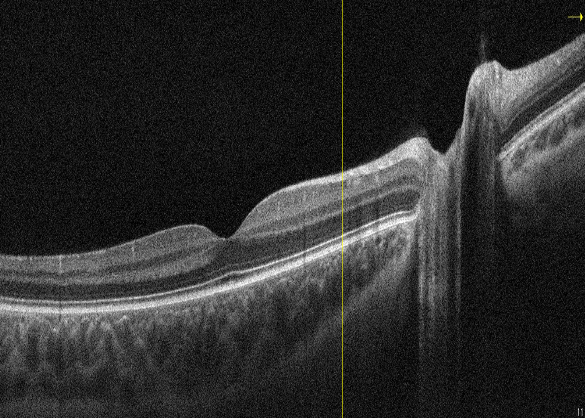  S5_Fig | 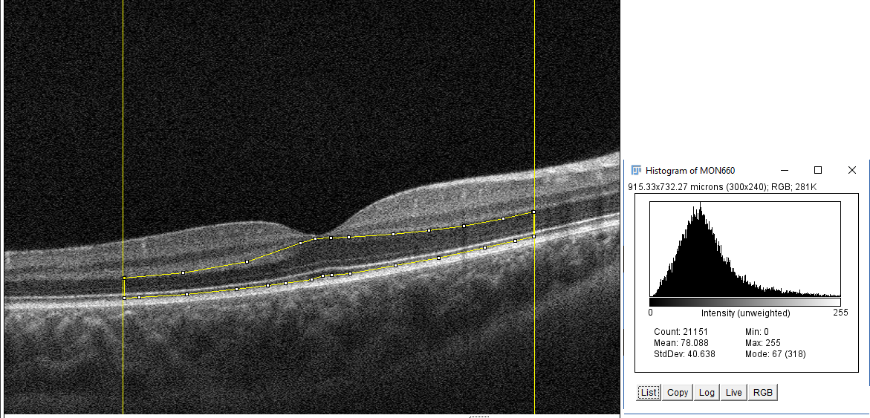  S6_Fig |
| 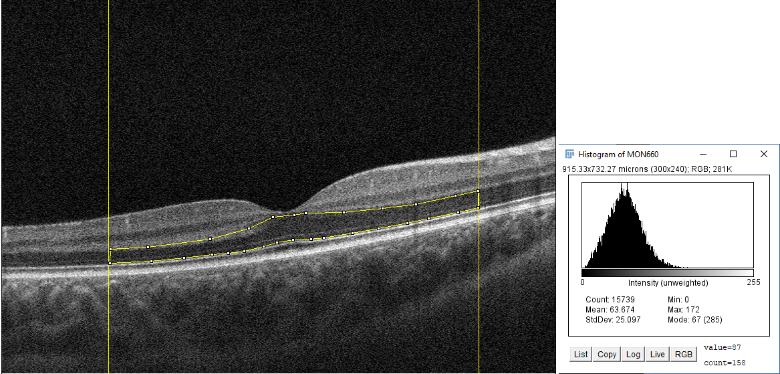  S7_Fig | 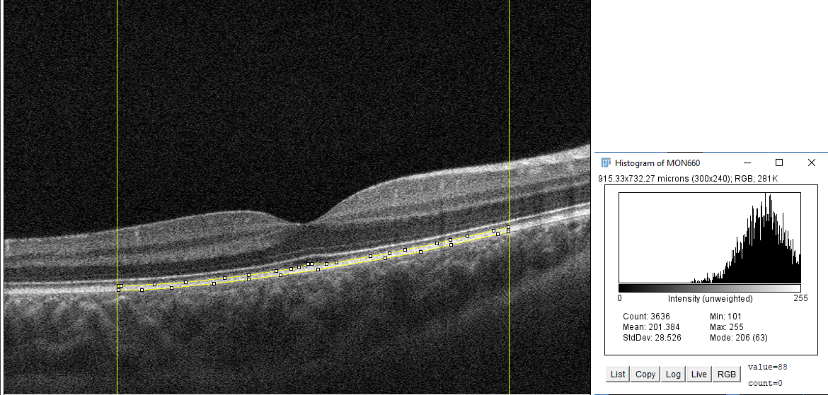  S8_Fig |
| 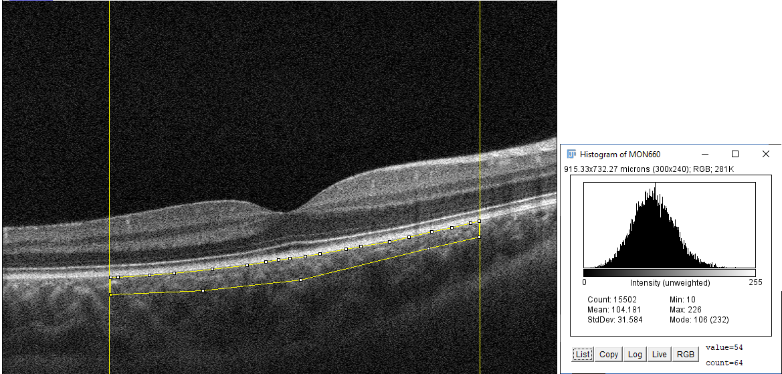  S9_Fig | 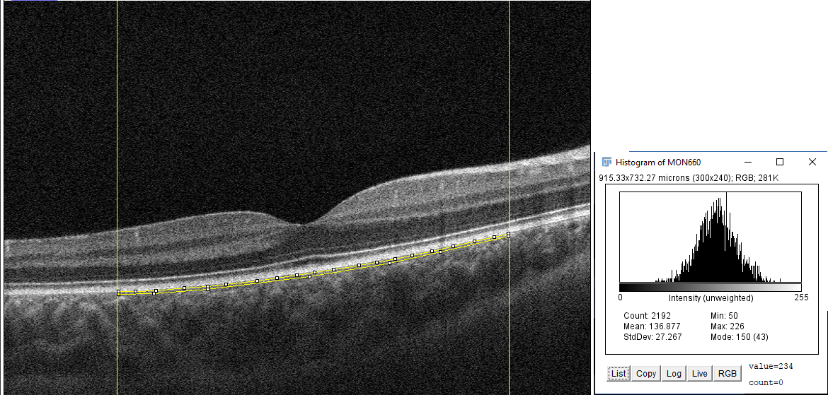  S10_Fig |
| 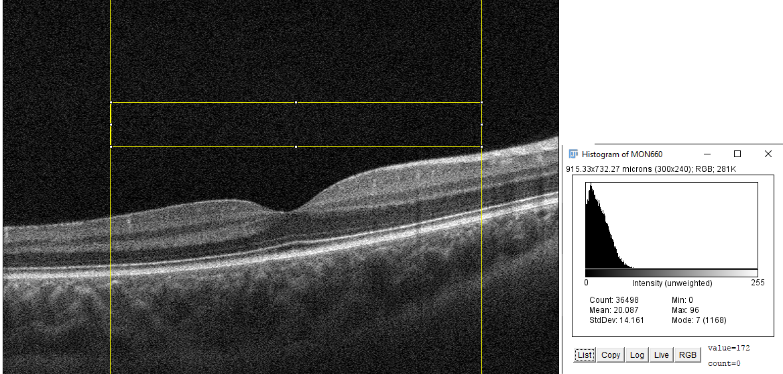  S11_Fig | 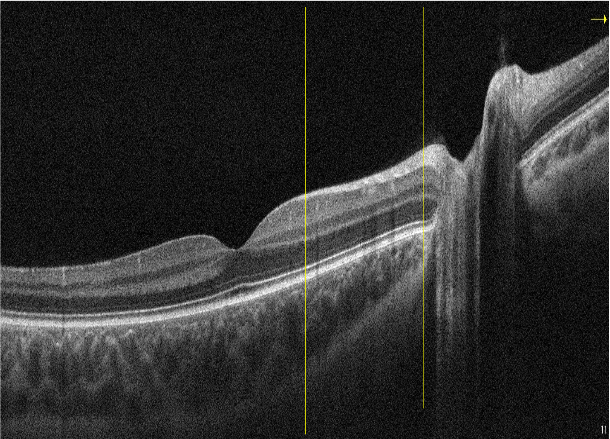  S12_Fig |
| 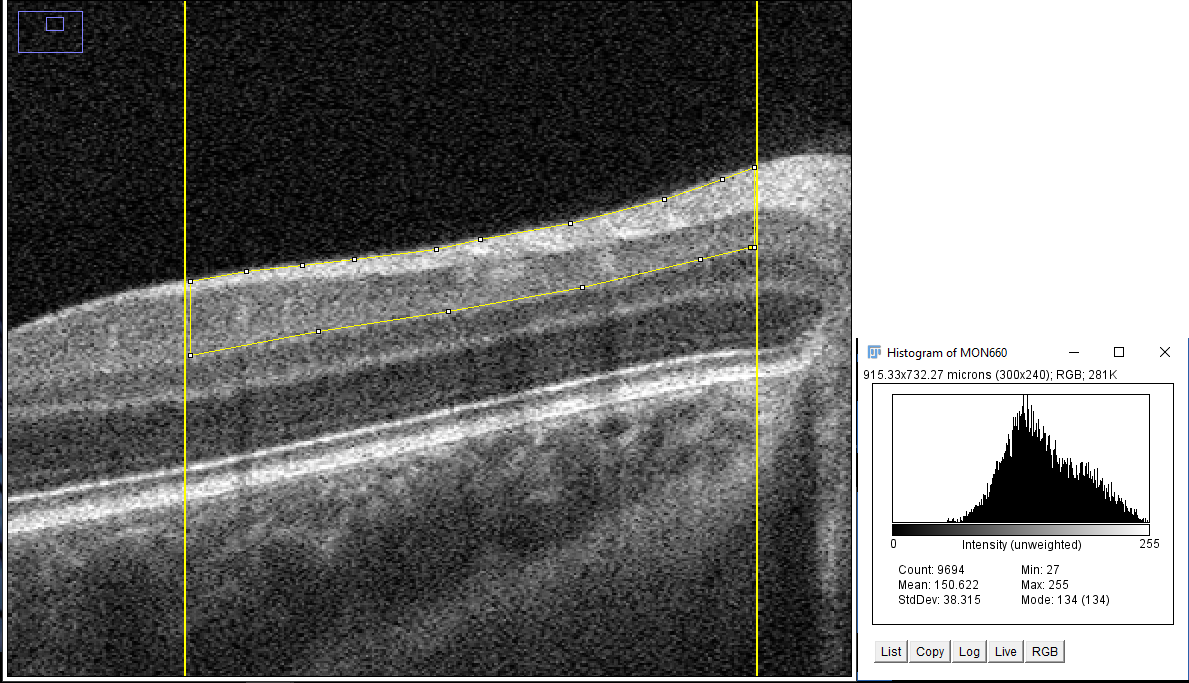  13_Fig | 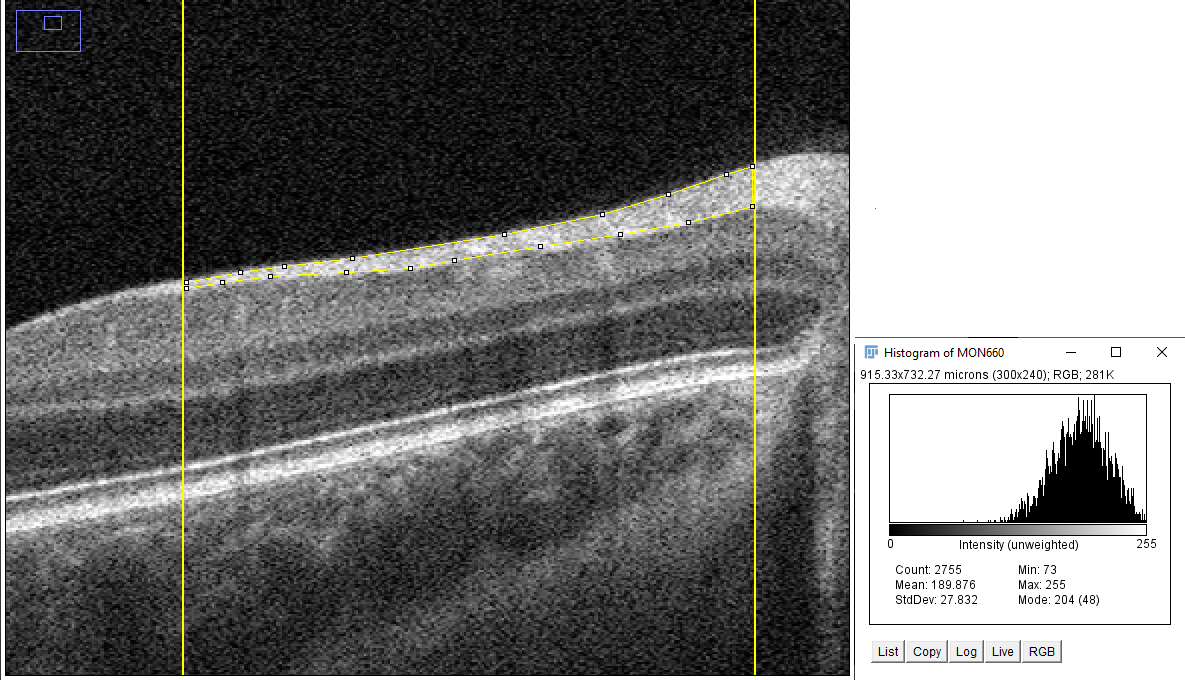  S14_Fig |
| 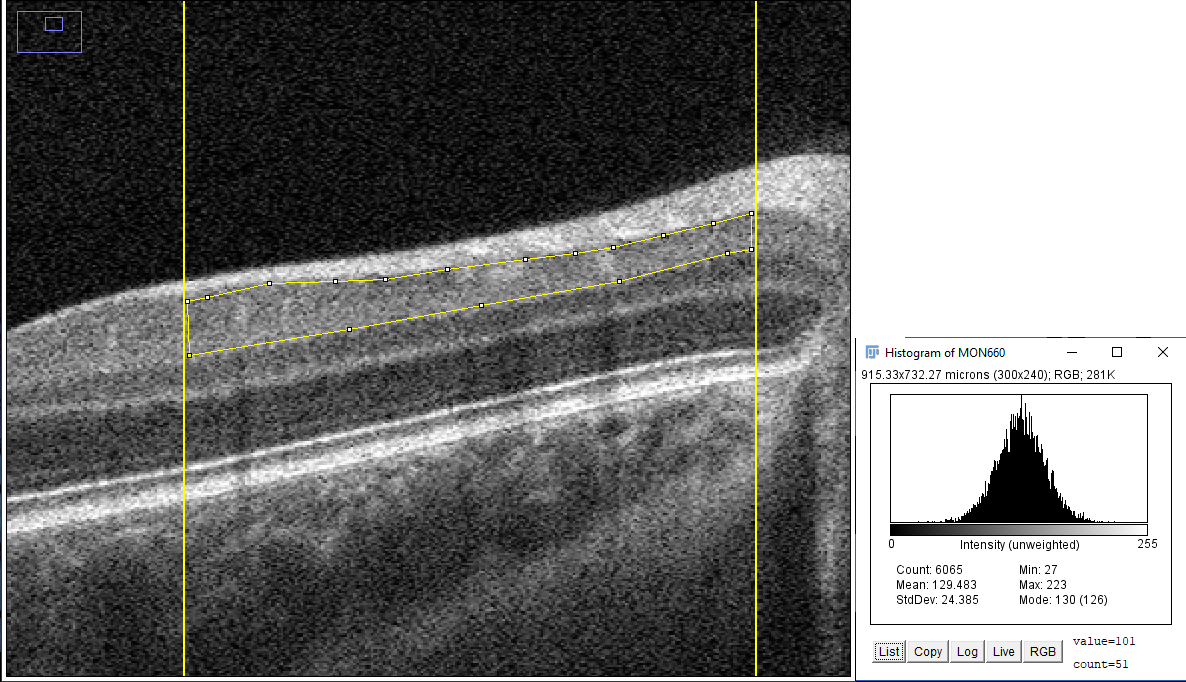  S15_Fig | 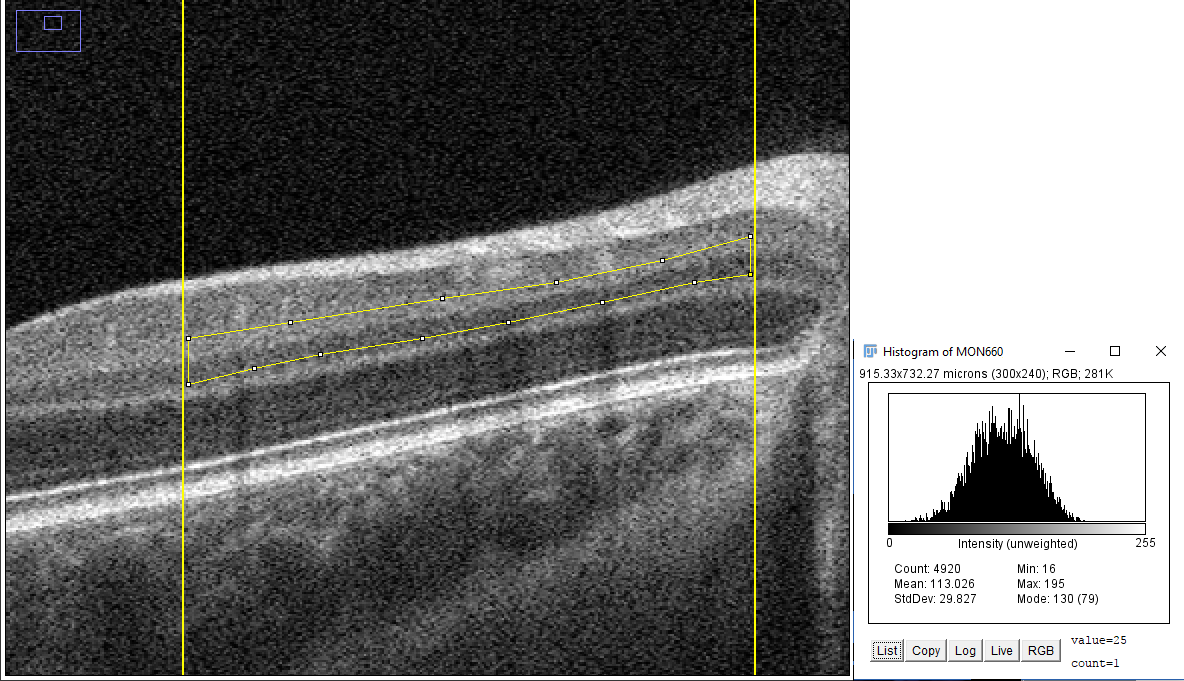  S16_Fig |
| 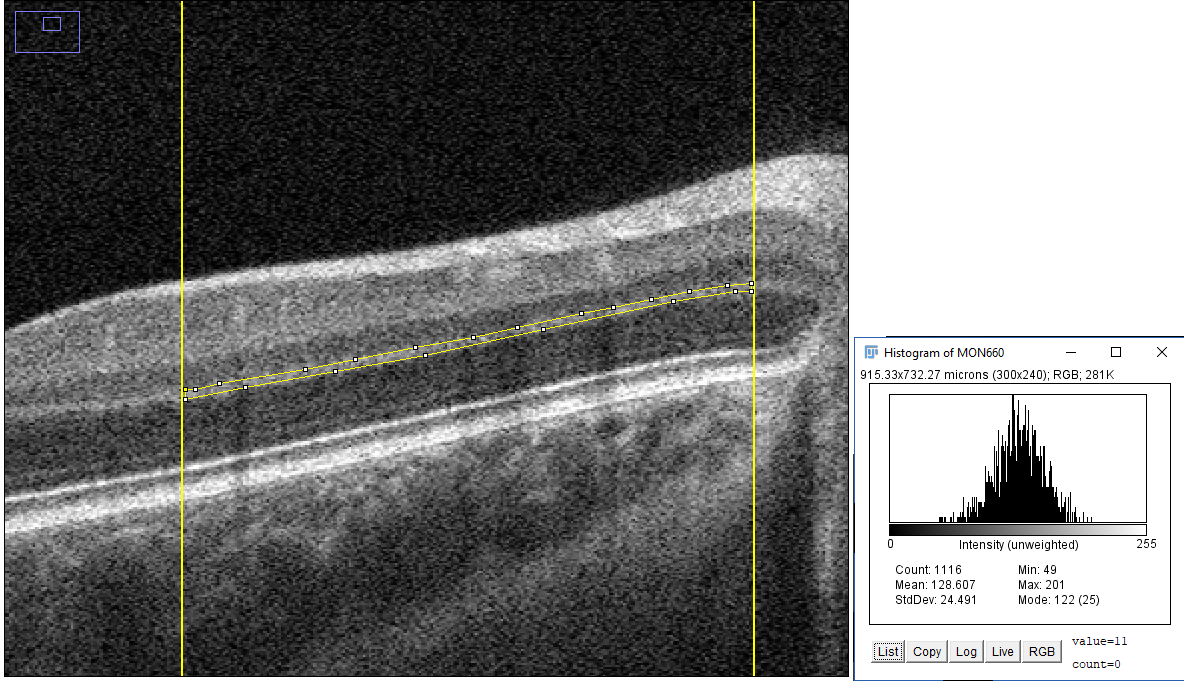  S17_Fig | 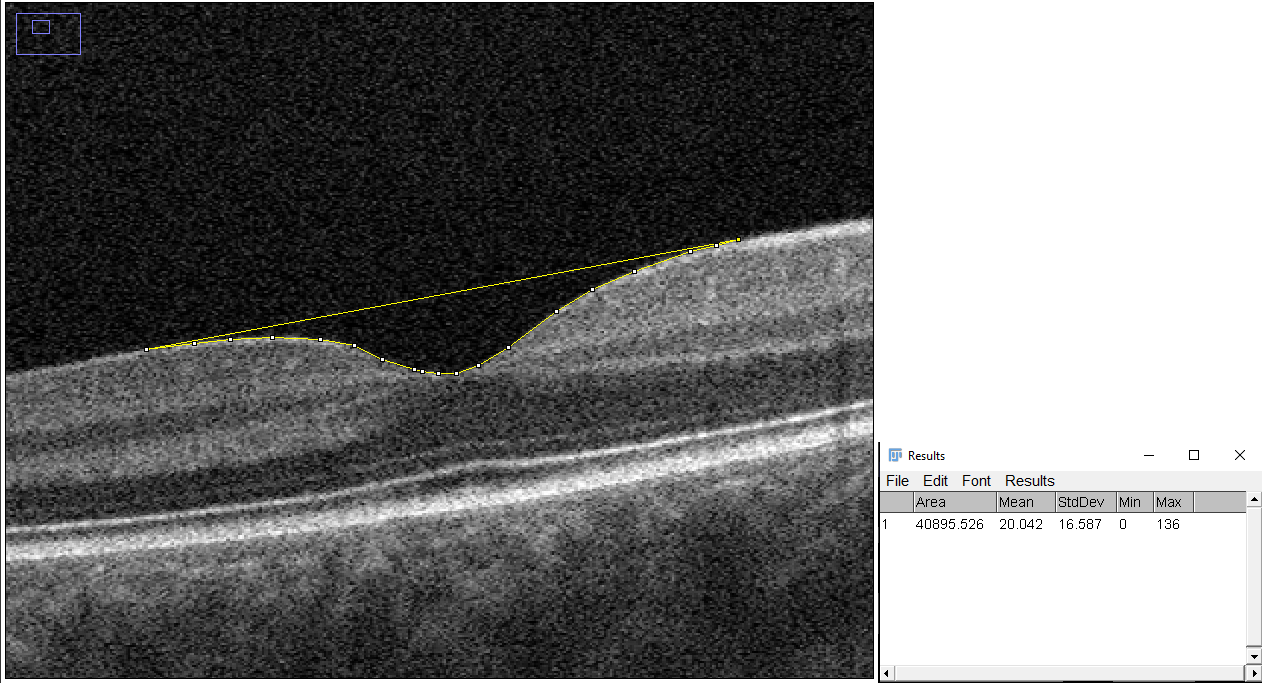  S18_Fig |
